# Dental Care Provision during Coronavirus Disease 2019 (COVID-19) Pandemic: The Importance of Continuous Support for Vulnerable Patients

**DOI:** 10.3390/medicina56060294

**Published:** 2020-06-12

**Authors:** Arkadiusz Dziedzic, Marta Tanasiewicz, Monika Tysiąc-Miśta

**Affiliations:** 1 Department of Conservative Dentistry with Endodontics, Medical University of Silesia, 40-055 Katowice, Poland; martatanasiewicz@sum.edu.pl; 2 Department of Prosthodontics and Dental Materials, Medical University of Silesia, 40-055 Katowice, Poland; mtysiac-mista@sum.edu.pl

**Keywords:** high-risk groups, COVID-19/SARS-CoV-2, coronavirus, patient-centered approach

## Abstract

As a result of the ongoing 2019 coronavirus disease (COVID-19) pandemic, the medical and dental services across the world have to tackle unprecedented situations, providing essential care and professional support. The global health care crisis caused directly by the vast number of severe COVID-19 cases, and indirectly by reduced access to health care, as well as by limited secondary care provision, had a major impact on specialist services, and subsequently the deterioration of medical and dental conditions, particularly in vulnerable persons. In particular, at present, special care dentistry seems to play a unique role, dealing with a wide range of patients with underlying medical conditions and co-morbidities, phobic individuals, and persons with learning/physical disabilities. The effective adaptation of health services to the current new reality, based on an empathetic approach and recent guidelines, would allow us to maintain an adequate care provision, minimizing the long-term impact of the pandemic.

As the dental care provision across the world was dramatically suspended in recent months, reduced to mainly emergency cases, in order to comply with current guidelines, the access to primary and secondary care is becoming increasingly more challenging [1,2,3]. Whilst some governments introduced strict ‘shielding’ measures to protect individuals, this turns out to be much more problematic for vulnerable patients, especially those with special needs who require additional capacities and facilities. The health complications associated with the 2019 novel coronavirus disease (COVID-19) are more presumable in the diverse high-risk group of population classified as ‘vulnerable’ due to their medical, physical, intellectual, and learning disabilities. It has to be noted that a vast majority of persons with special (care) needs are also vulnerable and, what is more, the recently released epidemiological data indicates that persons with a high Body Mass Index (BMI) score (obese), as well as ethnic minorities, are at a higher risk of contracting COVID-19 and subsequent serious health complications [4,5].

When facing the global crisis in health care due to the COVID-19 pandemic, we should ask ourselves how we can continue the provision of dental services, maintaining their adequate level, despite the displacement of routine clinical care. As a profession, above all, we have the duty to provide care, we are obliged to help our patients and never turn them away, regardless their medical status [6]. When looking forward and predicting the next recovery phase, it is paramount to be well prepared for the overflow of vast numbers of patients when restrictions are lifted. This is a predicted consequence of unfinished treatment plans, deferred routine care, suspended sedation/dental general anesthesia procedures, and accumulated cases of untreated odontogenic conditions. Questions arise as to how dental services can optimize primary and secondary care for our patients who are confused and scared, as well as how efficiently special care dentistry (SCD) can adapt to the present challenges, which result from the real danger of the Severe Acute Respiratory Syndrome Coronavirus 2 (SARS-CoV-2) pandemic.

## 1. Anticipated Deterioration of Medical and Dental Conditions

It is not surprising that a vast number of patients with complex needs may suffer from further medical and/or dental problems as a result of postponed medical/dental care, rescheduled surgeries, interrupted medication regimes, and a lack of access to consultations with general medical practitioners and specialists [7,8]. In the United Kingdom, people who are clinically extremely vulnerable and are at the greatest risk of developing serious complications as a result of COVID-19 received a letter advising them to shield for several weeks. The identified groups of extremely clinically vulnerable people who are at high risk of getting seriously ill from SARS-Cov-2 infection are presented in Table 1 (Public Health England, London, UK, [9]). This includes, clinically, people living in long-term care facilities for the elderly or people with special needs. It is confirmed that immunocompromised patients, such as persons undergoing anticancer therapy, those on chemotherapeutic drugs, transplant recipients, patients with underlying medical problems and comorbidities can be directly affected by the current situation, as this group of patients is undoubtedly highly vulnerable to coronavirus SARS-CoV-2 infection and subsequent health problems, including life-threatening ones [10].

Despite the non-coherent evidence of the direct relationship between impaired immune host status and serious COVID-19 complications, individuals with impaired immunity should be considered the greatest risk group. This category of patients should remain under the highest risk group label due to the severe progression of the illness. Similarly, dental patients who suffer from chronic autoimmune and/or inflammatory conditions such as pemphigus, Sjögren syndrome, or lupus erythematosus and who are undertaking long-term immunomodulatory therapy (using corticosteroids) are also predisposed to severe COVID-19 progression due to a reduction in host antiviral mechanisms [11]. These persons must be encouraged and advised to continue their therapy during the pandemic, as the potential exacerbation of the autoimmune-related oral lesions is likely to occur.

Apart from primary severe complications within the lower respiratory tract, according to the latest studies, SARS-Cov-2 appears to reveal neurotropic and cardiotropic affinity [12,13], with a potential impact on cognitive function and cardiovascular potency. In addition, impaired access to some medications and secondary care may expose medically compromised patients with complex health conditions to further health problems and subsequent complications [10,14]. As a result, the most common chronic diseases such as diabetes, hypertension, and heart conditions may become poorly controlled, affecting the general health status and restricting indications for more complex and invasive dental care. Interestingly, recently patients with lupus complained about the shortage of hydroxychloroquine in the US, a medication which is used to treat autoimmune conditions, among others, as all its supplies were redirected to hospitals [15]. Moreover, patients who have been receiving systemic pharmacotherapy with angiotensin-converting enzyme (ACE) inhibitors, as SARS-CoV-2 revealed a certain predilection to angiotensin-converting enzyme 2 (ACE2) receptors, can be potentially more susceptible severe respiratory tract complications resulting from COVID-19 [16,17]. On the contrary, the European Medicines Agency advises continued use of medicines for hypertension, heart or kidney diseases during the COVID-19 pandemic [18]. Similar problems may apply to corticosteroids prescribed to suppress the immune reactions in the case of asthma; however, the recommendations of professional bodies strongly support the advice for such patients to continue taking their regular medicines in line with their personalized asthma action plan [19].

## 2. Reduced Access to Secondary Dental Care and Fear Factor

The shortage of hospital and specialist staff and resources, as well as the cancellation of routine secondary dental care appointments led to a significant reduction in dental care under sedation and general anesthesia globally. Due to the risk of SARS-Cov-2 aerosol exposure, the use of fast-speed handpieces and three in one syringes, (ultra)sonic scaling, surgical extractions and minor oral surgery procedures involving bone structure removal cannot be carried out without enhanced personal protective equipment (PPE) [3,20,21]. Undoubtedly, the range of dental treatment under conscious sedation is to be drastically reduced, primarily affecting children and individuals with high levels of dental anxiety, requiring anxiety management measures. New SCD guidelines launched by the Royal College of Surgeons of England recommend that non-urgent treatment must be deferred to minimize risk to patients and staff, advising to involve two clinicians in the decision-making process. A wider use of various sedation techniques, instead of general anaesthesia, such as intravenous sedation as a safe and cost-efficient option will secure SCD provision during COVID-19 outbreak (Royal College of Surgeons (RCS), London, UK [22]). This is particularly valid nowadays because of suspended dental treatment under general anesthesia in hospital settings due to the prioritization of severe COVID-19 cases.

**Figure 1:**
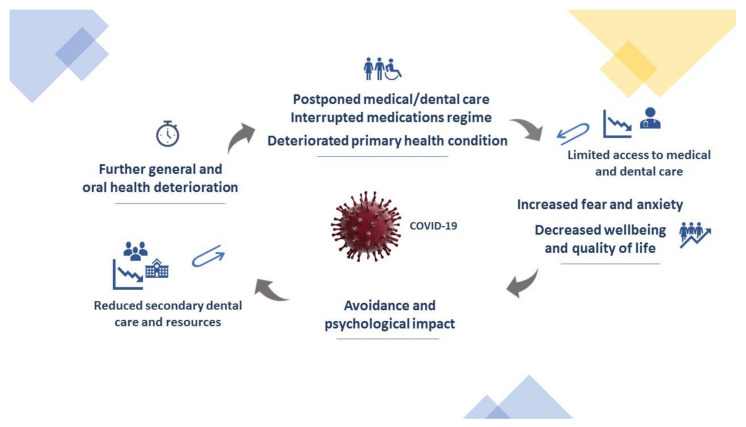
Accumulative effects of health and service provision problems during 2019 coronavirus disease (COVID-19) lockdown, leading to a vicious circle of further consequences, with the substantial role of anxiety, health deterioration and suspended routine medical/dental services.

Moreover, it is not difficult to imagine that the level of general anxiety in patients with mental health problems could be elevated at present, which would directly contribute to the limited dental care provision dedicated for phobic individuals [23,24]. It is likely that the number of acute mental disorders is going to be higher. Reference centers for tertiary and secondary care, such as hospitals, academic centers and community specialist clinics already implemented strict measures for accepting/rejecting referrals, with a knock-on effect on existing waiting lists (Figure 1). Consequently, these patients, eventually, are expected to return to primary care services, hoping to receive their planned care.

Specialist emergency care, including intravenous sedation and/or dental general anesthesia, must be rearranged somehow for those patients in constant pain who simply cannot wait any longer and for whom urgent antimicrobial and analgesic measures were not effective. As a result, the diversification and/or increasing use of non-pharmacological pain and anxiety control methods needs to be encouraged and promptly implemented. Dental teams, including SCD teams, should expand a range of various techniques and alternatives to pharmacological sedation or general anesthesia [25]. Unfortunately, vulnerable groups of elderly individuals who decided to implement a ’shielding’ strategy, staying at home for weeks, and unregistered patients with poor oral health and neglected dentition may especially face more difficult access to any kind of dental service at present. New strategic prophylactic programs on a national scale should be promptly introduced for these groups of vulnerable persons, as the dental care sector expects oral health deterioration and the increased incidence of oral diseases in the near future. On the contrary, this emphasizes the importance of prophylactic measures, prevention, regular dental assessments, follow-ups and maintenance, as persons who present with good and stable oral health usually do not require any special attention during a lockdown period.

## 3. Impact on Patients and Dental Team Safety

In light of the current COVID-19 pandemic, the principal message “be professional, stay safe, and be helpful” is particularly valid and up to date at present, as dental teams should at least continue to deliver emergency services equipped with strategic plans, supported by guidelines, and appropriate PPE. When compared to the medical workforce based in intensive care units, the dental sector is already in a reasonably safe position, as long as it follows the basic recommendations provided worldwide by different bodies and organizations (Centers for Disease Control and Prevention, World Health Organization (CDC/WHO). Afterall, from a cross-infection point of view, we should always protect ourselves and our patients, being well prepared against any infectious threat in dental surgery, according to the basic rule “treat every patient in the same professional manner”. The COVID-19 crisis shed light on aerosol generation during medical/dental procedures, including the assessment or treatment of potentially infectious cases. Moreover, it is likely that there will be a reorganization in the delivery of dental services, as there will be a requirement to use measures to contain the spread of the infection in dental surgeries permanently. This seems to be the most appropriate time to reflect on the current clinical standards associated with aerosol generating procedures, with a view to reconsidering or revising them, and clinicians need to gain a clear insight into how special care dentistry maybe delivered safely in the immediate and more distant future, in light of COVID-19. Furthermore, if a patient with special needs requires a ‘chaperone’ to be present during their appointment due to physical or intellectual disability, it is compulsory to provide the accompanying person with standard protective means, including an apron, standard surgical mask and/or shoe covers to maximize the reduction in the exposure to aerosols within the dental surgery.

SARS-CoV-2 coronavirus can be transferred via saliva [26] and, while a vaccine is not available, the standard PPE kit is fully applicable for non-aerosol-generating procedures (non-AGPs); however, it must be modified for aerosol generating procedures (AGPs) in dentistry that require higher than standard levels of protection, with the use of enhanced and additional PPE [3,20,21]. According to the most recent recommendations, due to the fact that rapid tests are not available, risk assessment is crucial, prior to performing even an extraction or intraoral radiographs, as these procedures can be potentially aerosol generating [3], (Table 2). Individualized risk assessments for persons with various conditions, for instance, immunosuppression, terminal oncological care and chronic pulmonary obstructive disease, should be central to clinical care. It must be emphasized that professional organizations may launch different, stricter guidelines regarding PPE [27]. Thus, every COVID-19 asymptomatic patient needs to be appropriately triaged and managed with local urgent dental care arrangements, prior to making decision about using PPE. Where possible, for COVID-19-positive persons, specifically designed dental centers to secure their oral health, with additional cross-infection preventative facilities, should be established.

It can be deduced that, for specialist dental services, standard fluid-resistant masks, long-sleeved gowns/aprons and well-designed visors are adequate to carry out non-AGP and to treat asymptomatic individuals when their COVID-19 status is unknown. Whilst some dental emergencies are considered AGP when high-speed rotors are used, the dental team would be required to wear enhanced PPE, including a surgical gown, filtering facepiece respirator (FFP3/N99), eye protection, a visor and hat, if the dental treatment was deemed to be an AGP or even if there was an increased risk that, due to procedural complications, a non-AGP clinical session may turn into an AGP one (Table 2). Risk assessments and pre-treatment planning are, as always, essential. In dental surgery, basic rules to mitigate aerosol generating, such as avoiding three in one syringes, the use of dental dams and high-volume suction, leaving a surgery for at least 30 min after AGP, using negative pressure facilities and booking patients who require emergency AGP at the end of the clinical session, might significantly reduce the risk of viral human-to-human transmission [1,3,24].

Due to the fact that SARS-CoV-2 is most present in the nasopharyngeal region and saliva/oropharyngeal secretions are the main reservoir of the virus [26], which can be transmitted via droplets, the implementation of preoperative measures is suggested in order to reduce the viral load inside the oral cavity, but they can be easily introduced as standard protocol. Pre-operational (pre-procedural) antimicrobial mouth rinse is generally believed to lower the amount of oral microbiota and it is suggested as an effective agent to reduce the risk of aerosol microbiological contamination in dental settings. The antimicrobial efficacy of common mouthwashes depends upon the concentration of active ingredients and their acidity, as low saliva pH might accelerate virulence through endocytic entry into the host cell [28]. It has been reported that SARS-CoV-2 is susceptible to oxidative compounds, and thus, subsequently, a pre-procedural mouth rinse containing oxidative agents such as those containing diluted hydrogen peroxide (1–2%), or 0.2% povidone iodine (PVP-I) are recommended [29,30]. Although the efficiency of other biocidal/viricidal intraoral measures such as topical swabbing and local disinfection is under investigation, only limited data is available in this respect. The exact mechanism is unknown, however it can be linked to the fact that the SARS-CoV-2 lipid ‘envelope’ can be disrupted when exposed to ingredients commonly found in mouthwashes, such as hydrogen peroxide and ethanol, as well as PVP-I and cetylpiridine [31]. Ethanol-based mouthwashes, with relatively low alcohol content that ranges from 14% to 27% can be potentially efficient against enveloped viruses, preventing subsequent viral replication; however, further research is urgently required to warrant their efficacy. In needs to be noted that PVP-I use can be contraindicated in the case of iodine hypersensitivity, pregnancy and severe thyroid disfunction, and also, in view of the alcohol-related risk of oral cancer, the clinicians should provide their patient with adequate advice. In practical scenarios, the use of pre-treatment mouthwash seems to be prudent in the current COVID-19 circumstances and, alternatively, the patient can be also advised to rinse oral cavity prior to attending dental appointment.

In addition, new inventions such as external portable high-volume high-efficiency particulate air (HEPA) filter technology or high-volume suction intraoral adapters used chairside can contribute to a vast reduction in aerosols released in clinical settings during dental assessment, diagnostics and/or treatment. Furthermore, minimizing staff exposure to potentially COVID-19 infected (but asymptomatic) patients, by implementation of pre-procedural point-of-care (POC) rapid and accurate molecular testing [32] for SARS-CoV-2 in SCD clinics, would support risk assessments, clinical management and save resources.

## 4. Time for Change and Adaptation for Dental Services

Amid all this confusion and uncertainty, the attitude of reassurance, accurate professional advice and appropriate care provision—even if this is restricted emergency and urgent cases only—are still the core elements of good dental practice and an ethical approach [33]. Dental teams are required to be creative, practical, and patient-minded, as the COVID-19 crisis underpins the shift from a repair model of care to a wellness-based model. Whilst the traditional ‘repair’ model of care concentrates strictly on solving a primary health problem ‘ad hoc’, the recovery wellness model of care provides a rationale for a long-term and permanent positive outcome, focusing on a holistic, person-centered approach. This model equally demonstrates health/dental care staff’s understanding and compassion while providing care, and also offers psychological support. We believe that being not only a competent, but also a truly empathetic clinician, is our main role in society. The idea of a patient-centered approach has gained its true definition, as everyone must be treated in a special way, while also taking into consideration their COVID-19 status. Clinicians dealing with vulnerable patients with underlying medical conditions should appreciate the value of AGP risk assessment in the informed care pathways in contemporary special care dentistry. On the other hand, there is a substantial impact of individualized feedback on their own oral health care. This is coherent with the most recent Public Health England initiative, named ‘Making Every Contact Count’, to provide support for patients to make positive behavior changes to their health and wellbeing [34], with its aim being to reduce the prevalence of long-term diseases in the population that are associated with behavioral and modifiable risk factors.

Despite some controversies, remote advice and ‘teleconsultations’—sometimes underestimated—underpin the management of patients who may feel abandoned or less cared for. Online consultations arranged utilizing patients’ self-described symptoms and photos provided by themselves could undoubtedly be an essential element of patient support. A plain conversation and professional advice, including oral hygiene and maintenance issues, may vastly contribute to the patient’s wellbeing, or to preventing the deterioration of oral health. For patients who suffer from chronic or acute oral mucosa diseases, basic advice, including the consumption of a balanced diet and nutrition, a reduction in smoking and alcohol, following the doctors’ instructions, and continuing prescribed pharmacotherapy, along with maintaining a positive mood and an appropriate level of exercise, should constitute the core professional recommendations. Nevertheless, these individuals often require a differential diagnosis, which might be crucial from a triaging point of view, as some of oral medicine cases are associated with a fever/elevated temperature, symptoms that are difficult to distinguish from respiratory tract infections [35]. Virtual consultations can be particularly valuable to arrange a ‘best interest meeting’ with peers involved in the care of a person who lacks capacity.

It is worth noting that, by establishing more flexible dental care environments, through adapting or expanding the existing facilities, dental sectors can quickly adjust to the current demands. Dental teams would welcome the involvement of professional bodies, including the International Association of Special Care Dentistry (IASCD) and the British Society of Disability and Oral Health (BSDH) in the provision of up-to-date recommendations for SCD specialty, supporting secondary health care during the COVID-19 crisis. The most recent guidance for SCD during the pandemic, provided by the Royal College of Surgeons (RCS, London) of England, is an excellent example of professional support. With this in mind, a significant reorganization of SCD might be required, accompanied by investments, to address the COVID-19 crisis at adequate levels of safety, considering the high risk, and complex medical profile of patients with special needs.

## 5. Empathy and Leadership above All

Due to the unprecedented extent of care provision suspension during the COVID-19 pandemic, the ‘empathy scale’ needs to be even broader, leading to a better understanding of patients’ problems that goes beyond standard practice. Dental services and service providers must support, devise, execute, and opt for difficult choices concerning priority patients, and give their patients confidence that the decisions taken are correct. Above all, we must show genuine empathy in light of the difficulties our patients face, and resolve their dental problems promptly, without any delay. Even clinically well-justified remote antibiotic or analgesic prescriptions faxed to local pharmacies may not be sufficient in the long term and, more importantly, this emergency protocol may significantly contribute to antibiotic resistance and/or other systemic complications. As a direct result of the reduced availability of dental care and the difficulty in accessing dental services due to deferred routine care during the COVID-19 pandemic, there is a significant risk of accidental analgesic overdose, particularly in self-isolating persons who try to self-manage their acute symptoms, with the use of over-the-counter acetaminophen (paracetamol) and nonsteroidal anti-inflammatory drugs (NSAIDs).

The more organized and supportive we are now on all dental service fronts, the more likely it is that the lockdown measures for routine dentistry are lifted earlier. We need to send a clear message that we will do everything we can to protect patients, while, on the other hand, patients should understand and try to do all they can to protect all of us. As we know, in some parts of the world, the ‘coronavirus plan’ has been working well because clinic leads, hospital managers, owners of dental practices and academic staff heads behave like true leaders and appear to be capable of tackling the health crisis. Leaders should do all that is feasible to help dental services get through the pandemic and not leave any patient without appropriate care. This can be achieved by service conversion, adaptation to the anticipated demands of specialist procedures in future, and utilizing basic infection control measures, including advanced PPE for all staff involved in dental care, if clinically justified. In addition, promoting online consultations and advice, along with the broader use of recovery wellness and patient-focused models are easily applicable by any service, including primary medical care (general medical practice), mental health care, and most specialist secondary services. So-called rapport building, a technique used to manage anxious patients that involves, i.e., sending a letter to the patient, would be equally supportive for reassurance and advice purposes. We are convinced that the partial transition from a general anesthesia-based model to the more common use of sedation methods (intravenous, transmucosal) in outpatient clinics can be implemented by minor surgery units during the pandemic, including otorhinolaryngology gastro-enterology and dermatology disciplines. In the coming months, we need to take the opportunity to reflect on the challenge of the COVID-19 pandemic crisis. As the wellbeing of health professionals is going to be inevitably affected, it will be necessary to care not only for those people who need care, but also those who deliver it.

## 6. Conclusions

By providing safe dental care with the use of dedicated special measures, and redefining our role, special care dentistry services are obliged to meet the oral health needs among those who are the most vulnerable and who are at risk of health decline. Primary and secondary services must maintain an appropriate level of clinical activity by reducing inequalities related to the access to specialist dental care. The most compromised patients expect continuous support, with the provision of—at the very least—emergency dental care that is fit for purpose, verifying the preparedness of dental providers. In particular, at present, special care dentistry seems to play an ever more important role, dealing with a wide range of patients with underlying co-morbidities, phobic individuals, and persons with learning/physical disabilities. The effective and rapid adaptation of health services to the current new reality, based on an empathetic approach and recent guidelines, will allow for adequate and safe care provision.

## Figures and Tables

**Table 1:** The list of clinically extremely vulnerable groups based on expert opinions and evidence-based data (Public Health England UK, government source [9]).

Categories of Medically Compromised People at Greatest Risk of COVID-19 Serious Illness
Solid organ transplant recipients; People with cancer who are undergoing active chemotherapy; People with lung cancer who are undergoing radical radiotherapy; People with cancers of the blood or bone marrow such as leukemia, lymphoma or myeloma who are at any stage of treatment; People having immunotherapy or other continuing antibody treatments for cancer; People having other targeted cancer treatments which can affect the immune system, such as protein kinase inhibitors or PARP inhibitors; People who have had bone marrow or stem cell transplants in the last 6 months, or who are still taking immunosuppression drugs; People with severe respiratory conditions including all cystic fibrosis, severe asthma and severe chronic obstructive pulmonary disease (COPD); People with rare diseases that significantly increase the risk of infections (such as severe combined immunodeficiency (SCID), homozygous sickle cell); People on immunosuppression therapies sufficient to significantly increase risk of infection; Women who are pregnant with significant heart disease, congenital or acquired; Other people have also been classed as clinically extremely vulnerable, based on clinical judgement and an assessment of their needs. GPs and hospital clinicians have been provided with guidance to support these decisions.

**Table 2:** Classification of dental procedures based on the risk of generating aerosol in dental settings (own proposal, based on Faculty of General Dental Practice, UK; modified and extended).

Non-Aerosol Generating Procedures	Aerosol Generating Procedures	Procedures Likely to Generate Aerosol
Basic dental examination Non-surgical, simple extraction of single-rooted mobile tooth Hand scaling with suction Caries removal using hand excavator Panoramic radiographs Temporary dressing without air–rotor cavity preparation Sutures and their removal Local anesthesia Full removable denture fit (final stage), which does not require adjustment Mouthguard fit Application of fluoride varnish within anterior quadrant, with the use of cotton roll for tooth surface drying	Use of high-speed handpiece (turbine) for any restorative procedures, opening and drainage, pulp chamber access, root canal treatment, etc. Use of a physiodispenser for surgical extractions, root sectioning, etc. Use of a high-pressure three in one syringe Use of ultrasonic scalers Maxillofacial and oral surgery with the use of piezosurgical devices Teeth polishing and brushing with slow-speed handpiece, air polishing Tooth structure preparation for fixed prosthodontics Implant installation	Intraoral radiographs Difficult extraction of molar tooth with compromised tooth structure The use of a slow-speed handpiece without suction Any procedure in patients with excessive saliva production when the use of suction is not possible Any dental procedure in, e.g., elderly patients who are prone to cough and who demonstrate a severe gag reflex Any dental procedure and significant behavioral issue which may increase the risk of AG (e.g., ADHD, ASD) Impressions for dentures, bridges, crowns, veneers and orthodontic appliances Oral swabbing
